# Mental Health Diagnoses Among Transgender Patients in the Clinical Setting: An All-Payer Electronic Health Record Study

**DOI:** 10.1089/trgh.2019.0029

**Published:** 2019-11-01

**Authors:** Jonathon W. Wanta, Joshua D. Niforatos, Emily Durbak, Adele Viguera, Murat Altinay

**Affiliations:** ^1^ Residency Training Program, Department of Psychiatry and Behavioral Sciences, University of Washington, Seattle, Washington.; ^2^ Residency Training Program, Department of Emergency Medicine, Baltimore, Maryland.; ^3^ Cleveland Clinic Lerner College of Medicine, Cleveland, Ohio.; ^4^ Department of Psychiatry, Neurological Institute, Cleveland Clinic, Cleveland, Ohio.

**Keywords:** electronic health records, mental health, psychiatric diagnosis, transgender

## Abstract

We performed a cross-sectional analysis of the prevalence of psychiatric diagnoses among transgender patients in clinical care using an all-payer electronic health record database. Of 10,270 transgender patients identified, 58% (*n*=5940) had at least one psychiatric diagnosis compared with 13.6% (*n*=7,311,780) in the control patient population (*p*<0.0005). Transgender patients had a statistically significant increase in prevalence for all psychiatric diagnoses queried, with major depressive disorder and generalized anxiety disorder being the most common diagnoses (31% and 12%, respectively). Utilizing an all-payer database, although not without limitations, enables assessment of mental health and substance use diagnoses in this otherwise small population.

## Introduction

There is a relative paucity of research related to mental health diagnoses in transgender persons. There has been clear and consistent data showing an increased risk for mood and anxiety disorders in transgender individuals, and there is emerging evidence looking at increased rates of bipolar disorder, post traumatic stress disorder (PTSD), and substance use disorders.^1–4^ Current estimates are based on small sample sizes and, often, self-report surveys. With a national prevalence estimated at 0.033–0.39%,^5,6^ obtaining a robust sample size to ascertain the prevalence of mental health disorders has proven difficult. Recent attempts at characterizing this population has utilized electronic health records (EHRs) to describe mental health and chronic diseases among Medicare beneficiaries as well as acute cardiovascular disease with use of gender-affirming hormone therapy.^7,8^ In this study, we sought to estimate the prevalence of mental health diagnoses among transgender persons using a cloud-based all-payer EHR database.

## Methods

Explorys, Inc. (Cleveland, OH) is a cloud-based EHR database comprised of ∼60 million unique patients from 26 U.S. health care systems across all 50 states.^9,10^ Data are uploaded every 24 h to the database where it is de-identified, standardized, and normalized to unified medical language system (UMLS) ontologies, including the Systematized Nomenclature of Medical (SNOMED) for clinical terms and Medical Subject Headings (MESH) for medical topics.^11,12^ UMLS searches allow researchers to use Explorys' web application PopEx to search through aggregated, de-identified population level data.

We conducted a retrospective observational cohort study utilizing Explorys to identify transgender patients with a history of a psychiatric diagnosis. Data were collected from 1999 to April 3, 2018. We identified transgender persons using the methods outlined by Progovac et al. for EHR studies,^7^ which included SNOMED ontology searches for gender minority-related International Classification of Diseases (ICD)-9 code (302.5, 302.50–302.53, 302.85, 302.6, 302.3) as outlined by McDowell et al.^13^ Aggregate, population-level data were then queried from this cohort for psychiatric diagnoses using SNOMED ontology searches in Explorys. Data were compared with the nontransgender (i.e., cisgender) patient population in Explorys. Demographic data are presented as numbers, percentages, and interquartile ranges. Chi-square tests and Fisher's exact test were used for inter-group comparison. Statistical significance was *p*<0.005 as recommended by Ioannidis when using large data sets.^14^ Analyses were performed using IBM SPSS Statistics, Version 25 (IBM). Use of Explorys has been deemed exempt from Institutional Review Board approval by University Hospitals Cleveland Medical Center.

## Results

We identified 53,449,400 patients, of which 10,270 (0.0192%) had gender minority-related codes. The median age was 35–39 years old for both groups (*p*=0.99). Table 1 lists the prevalence of *Diagnostic and Statistical Manual of Mental Disorders, Fifth Edition* (DSM-5) diagnoses for each population. Approximately 58% of transgender patients had at least one DSM-5 diagnosis compared with 13.6% of cisgender patients (*p*<0.0005). Transgender patients had increased prevalence for all psychiatric diagnoses queried (Table 1), with major depressive disorder and generalized anxiety disorder being the most common diagnoses (31% and 12%, respectively). There was also an increased lifetime prevalence of bipolar disorder (11%) and psychotic disorders (4.7% overall, 2.5% for schizophrenia and 2.2% for schizoaffective disorder) in transgender adults. Ten percent of transgender adults had a history of a substance use disorder, including 4.2% for alcohol and 3.8% for cannabis.

**Table 1.** Mental Health Diagnoses Among Transgender Patients and the General Population

Diagnosis	Transgender population	Control population
	*n*=10,270 (%)	*n*=53,449,400 (%)
Mood disorder	4720 (46)	4,802,280 (9.0)
Major depressive disorder	3210 (31)	2,549,270 (4.8)
Dysthymia	700 (6.8)	739,450 (1.4)
Bipolar disorder	1200 (11)	685,300 (1.3)
Anxiety disorder	3220 (31)	3,194,050 (6.0)
General anxiety disorder	1260 (12)	1104270 (2.0)
Panic disorder	460 (4.4)	393,690 (0.74)
Phobic disorder	300 (2.9)	77,830 (0.15)
Social phobia	220 (2.1)	29,860 (0.06)
Agoraphobia	90 (0.87)	43,660 (0.08)
Post-traumatic stress disorder	690 (6.7)	275,730 (0.52)
Obsessive compulsive disorder	210 (2.0)	113,200 (0.21)
Psychotic disorder		
Schizophrenia	260 (2.5)	196,820 (0.37)
Schizoaffective disorder	230 (2.2)	88,140 (0.16)
Adjustment disorder	830 (8.1)	631,320 (1.2)
Dissociative disorder	130 (1.3)	51,690 (0.10)
Somatoform disorder	100 (0.97)	96,410 (0.18)
Personality disorder	610 (5.9)	199,860 (0.37)
Cluster a personality disorder	20 (0.19)	6750 (0.01)
Cluster b personality disorder	350 (3.4)	62,660 (0.11)
Borderline personality disorder	320 (3.1)	47,390 (0.09)
Cluster C personality disorder	20 (0.19)	7700 (0.01)
Eating disorder	210 (2.0)	133,510 (0.25)
ADHD	1070 (10)	916,370 (1.7)
PDD	250 (2.4)	109,710 (0.20)
Autism	150 (1.5)	83,760 (0.16)
Chemical dependence		
Substance use disorder	1040 (10)	1,410,960 (2.6)
Alcohol	440 (4.2)	737,820 (1.4)
Cannabis	390 (3.8)	334,230 (0.63)
Cocaine	140 (1.4)	144,200 (0.27)
Opioid	120 (1.2)	157,150 (0.27)
Amphetamine	80 (0.77)	69,940 (0.13)
Tobacco user	2380 (23)	5,252,940 (9.8)
Percentage with at least one diagnosis	5940 (58)	7,311,780 (13.6)

^a^ *p* Values are from global *χ*^2^ and Fisher's exact tests.

^b^ Significance is set to *p*<0.005. All associations were significant unless otherwise noted.

ADHD, attention-deficit/hyperactivity disorder; PDD, pervasive developmental delay.

## Discussion

This is, to our knowledge, the largest investigation of mental health diagnoses and substance use prevalence in transgender individuals. We found a statistically significant increase in mental health diagnoses, including mood and anxiety disorders, PTSD, schizophrenia, personality disorders, attention-deficit/hyperactivity disorder, autism, and substance use disorders. This increased risk has been attributed, in part, to the high rates of discrimination and violence transgender individuals experience.^15^ The rates seen in this article are likely inflated by several confounding factors, including the prerequisite mental health assessment before starting medical interventions for transitioning, which may lead to misdiagnosis or overdiagnosis.

Although the results of this study suggest increased lifetime prevalence of all mental health disorders among transgender persons, the data do not allow us to track symptoms longitudinally over a patient's medical or surgical transition, which nonrandomized prospective and retrospective studies suggest improve mental health.^16^ The emerging field of transgender pediatric research has demonstrated improved mental health outcomes when children are allowed to socially transition at a young age.^17^ Improved social support in childhood among transgender persons may be a critical point of intervention for the prevention of significant mental illness in adulthood as described in this article.

This study was limited by not having access to individual patient charts through the Explorys database. Thus, we were not able to confirm gender minority-associated codes nor query transgender patients who had not formally received one of these codes in their EMR. In addition, there is a rich diversity of gender expression, and current ICD codes fall short, likely excluding a large percentage of transgender and gender nonconforming individuals from this analysis. Finally, we have no method through Explorys to verify the individual mental health diagnoses, the credentials of the health care worker assigning that diagnosis, when they might have been diagnosed, or how diagnoses may have changed over time. Ultimately, the sensitivity of a large database study such as this must be compared against smaller studies with a higher specificity.

## Abbreviations Used

ADHD: attention-deficit/hyperactivity disorder
EHR: electronic health record
ICD: International Classification of Diseases
SNOMED: Systematized Nomenclature of Medical
UMLS: unified medical language system

## References

[1] Heylens G, Elaut E, Kreukels BP, et al. Psychiatric characteristics in transsexual individuals: multicentre study in four European countries. Br J Psychiatry. 2014;204:151–156 23869030 10.1192/bjp.bp.112.121954

[2] Hepp U, Kraemer B, Schnyder U, et al. Psychiatric comorbidity in gender identity disorder. J Psychosom Res. 2005;58:259–261 15865950 10.1016/j.jpsychores.2004.08.010

[3] Beckwith N, McDowell MJ, Reisner SL, et al. Psychiatric epidemiology of transgender and nonbinary adult patients at an urban health center. LGBT Health. 2019;6:51–61 30707624 10.1089/lgbt.2018.0136 PMC6434596

[4] Dhejne C, Van Vlerken R, Heylens G, Arcelus J Mental health and gender dysphoria: A review of the literature. Int Rev Psychiatry Abingdon Engl. 2016;28:44–57 10.3109/09540261.2015.1115753 26835611

[5] Meerwijk EL, Sevelius JM Transgender population size in the United States: a meta-regression of population-based probability samples. Am J Public Health. 2017;107:e1–e8 10.2105/AJPH.2016.303578 PMC5227946 28075632

[6] Roblin D, Barzilay J, Tolsma D, et al. A novel method for estimating transgender status using electronic medical records. Ann Epidemiol. 2016;26:198–203 26907539 10.1016/j.annepidem.2016.01.004 PMC4772142

[7] Progovac AM, Le Cook B, Mullin BO, et al. Identifying gender minority patients' health and health care needs in administrative claims data. Health Aff (Millwood). 2018;37:413–420 29505378 10.1377/hlthaff.2017.1295 PMC5942884

[8] Darios Getahun, Nash R, Flanders D, Baird T, et al. Cross-sex hormones and acute cardiovascular events in transgender persons: A Cohort Study. Ann Intern Med. 2018;169:205–213 29987313 10.7326/M17-2785 PMC6636681

[9] IBM Explorys. Available at: https://www.ibm.com/watson/health/explorys Accessed 813, 2018

[10] Kaelber DC, Foster W, Gilder J, et al. Patient characteristics associated with venous thromboembolic events: a cohort study using pooled electronic health record data. J Am Med Inform Assoc. 2012;19:965–972 22759621 10.1136/amiajnl-2011-000782 PMC3534456

[11] U.S. National Library of Medicine. Overview of SNOMED CT. 2016 Available at: https://www.nlm.nih.gov/healthit/snomedct/index.html Accessed April 20th, 2018

[12] McDonald CJ, Huff SM, Suico JG, et al. LOINC, a Universal Standard for Identifying Laboratory Observations: A 5-year update. Clin Chem 2003;49:624–633 12651816 10.1373/49.4.624

[13] McDowell A, Progovac AN, Le Cook B, Rose S Estimating the health status of privately insured gender minority children and adults. LGBT Health Med. 2019;6:289–296 10.1089/lgbt.2018.0238 31314674

[14] Ioannidis JP The proposal to lower *p*-value thresholds to. 005. JAMA. 2018;319:1429–1430 29566133 10.1001/jama.2018.1536

[15] Winter S, Diamon M, Green J, et al. Transgender people: health at the margins of society. Lancet. 2016;388:390–400 27323925 10.1016/S0140-6736(16)00683-8

[16] Heylens G, Verroken C, Cock SD, et al. Effects of different steps in gender reassignment therapy on psychopathology: A prospective study of persons with a gender identity disorder. J Sex Med. 2014;11:119–126 24344788 10.1111/jsm.12363

[17] Olson KR, Durwood L, DeMeules M, McLaughlin KA Mental Health of Transgender Children Who Are Supported in Their Identities. Pediatrics. 2016;137:e20153223 26921285 10.1542/peds.2015-3223 PMC4771131

